# Supplementary figures and images for: Trisomy 21 induces pericentrosomal crowding delaying primary ciliogenesis and mouse cerebellar development
Source: eLife. 2023 Jan 19;12:e78202. doi: 10.7554/eLife.78202 (PMC9851619; doi:10.7554/eLife.78202)

**Figure 3-source data 2**

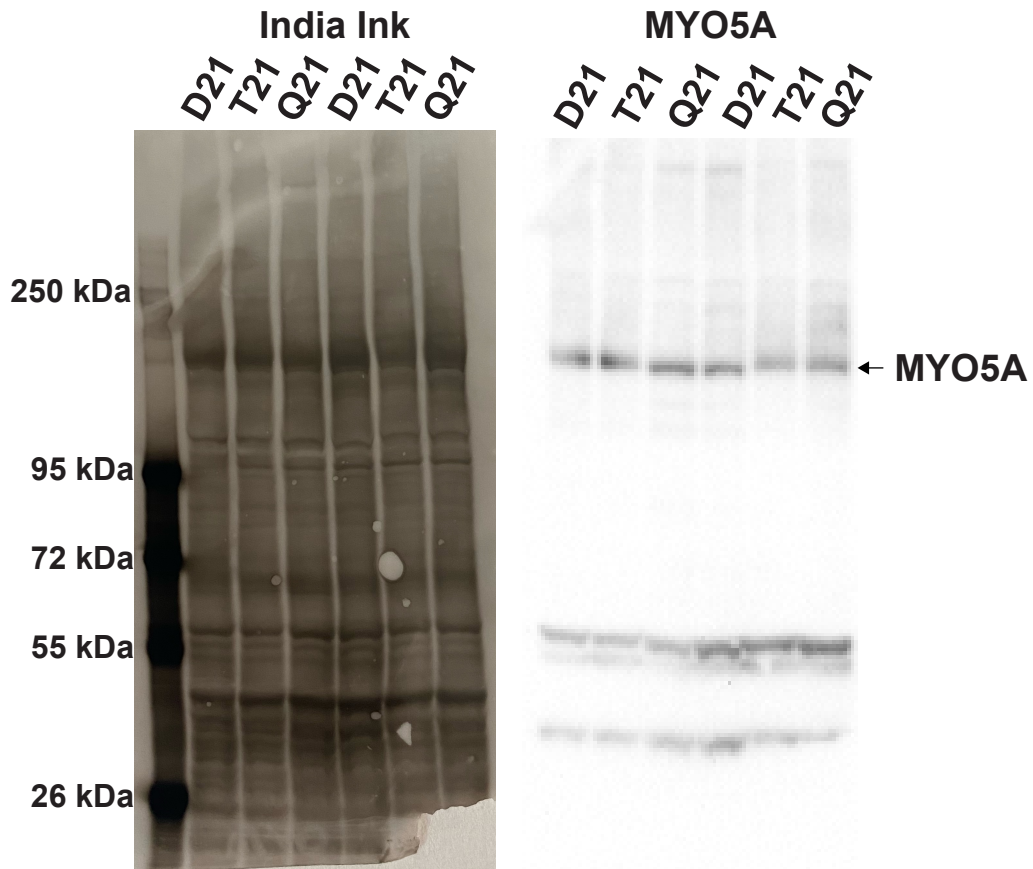

Supplement: Figure 3—figure supplement 1—source data 1. [file elife-78202-fig3-figsupp1-data1.pdf]

Figure 6-source data 2

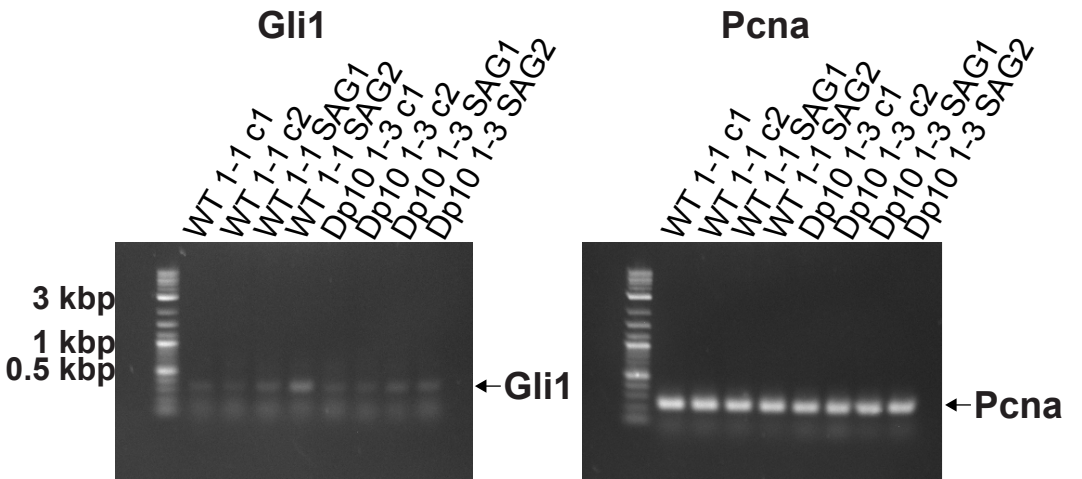

Supplement: Figure 6—figure supplement 1—source data 1. [file elife-78202-fig6-figsupp1-data1.pdf]
